# Parenchymal Brain Abscess as an Intracranial Complication After Sinusitis

**DOI:** 10.7759/cureus.17365

**Published:** 2021-08-22

**Authors:** Maria C Michali, Ioannis G Kastanioudakis, Lentiona V Basiari, Georgios Alexiou, Ioannis D Komnos

**Affiliations:** 1 Department of Otorhinolaryngology, Head and Neck Surgery, University Hospital of Ioannina, Ioannina, GRC; 2 Department of Neurosurgery, University Hospital of Ioannina, Ioannina, GRC

**Keywords:** acute sinusitis, brain abscess, intracranial complication, craniotomy, infection

## Abstract

Brain abscesses caused by sinusitis are rare in the antibiotic era. The purpose of the current manuscript was to report a rare case of a brain abscess located mainly in the frontal lobe after sinusitis, which was initially thought to be meningitis or encephalitis. A 39-year-old man was transferred to our hospital from another neighbouring hospital with tonic-clonic seizures, severe headache, and purulent nasal secretions. For one week, he was taking antibiotics for sinusitis. The computed tomography indicated lesions in the right sinuses but not in the parenchymal brain and thus antibiotics along with antiepileptic drugs were given. However, due to the deterioration of symptoms, magnetic resonance imaging was executed, which revealed an abscess in the frontal lobe. Afterward, an anterior ethmoidectomy and middle maxillary antrostomy were performed in order to drain the purulent content from the right sinuses. Ten days later, the patient presented disorientation and thus an open craniotomy for successful removal of the parenchymal abscess was performed. One month later, the patient was discharged with mild irritability, which was eliminated gradually over the next two months. Conclusively, brain abscesses can be caused by local spread from an infection of the paranasal sinus. The contribution of imaging modality is very significant not only for the early diagnosis but also for the therapeutic management of such cases. Frequently antibiotic treatment is insufficient and surgery may be required.

## Introduction

Brain abscesses are severe and potentially life-threatening intracranial infections that can be caused by hematogenous spread from a distant place or local spread from adjacent infected tissues. Regarding the latter, they have been referred to as later sinogenic complications in 10-20% of undiagnosed or misdiagnosed cases [1,2]. Especially, frontal sinusitis is considered as a predisposing factor for the occurrence of abscess in the frontal lobe due to proximal position [2].

However, in the last decades with the extensive use of antibiotics and diagnostic imaging tools such as computed tomography (CT) and magnetic resonance imaging (MRI), intracranial abscesses have been rarely reported [3,4]. The incidence of rhinogenic intracranial complications is still relatively high in geographically isolated regions and populations with socioeconomic problems, where medical care is problematic, or in cases of delayed diagnosis of paranasal sinusitis [1,3-5].

The purpose of our study was to present a rare case of brain abscess located mainly in the frontal lobe, which was caused by a contiguous spread from a sinus infection. We report the clinical manifestations of the pathology, the diagnostic process, and the management strategy that was followed in order to deal with this serious parenchymal brain complication.

## Case presentation

A 39-year-old male was transferred to our university hospital from a rural general hospital with generalized tonic-clonic seizures. The patient presented high fever, severe headache, and purulent nasal secretions over the last three days but his vital parameters were normal. Apart from his statement that he was a tobacco smoker, no previous medical conditions were reported.

Initially, one week prior to hospitalization, he was diagnosed with inflammation of the paranasal sinusitis by a private otorhinolaryngologist who had prescribed a combined antibacterial medical treatment of amoxicillin/clavulanate potassium and clindamycin. However, five days later, he was referred to the emergency department (ED) of the rural hospital with epileptic seizure. Regarding his medical history, the patient had suffered from sinusitis five years ago but no history of epilepsy was mentioned, not even in his family history.

**Figure 1:**
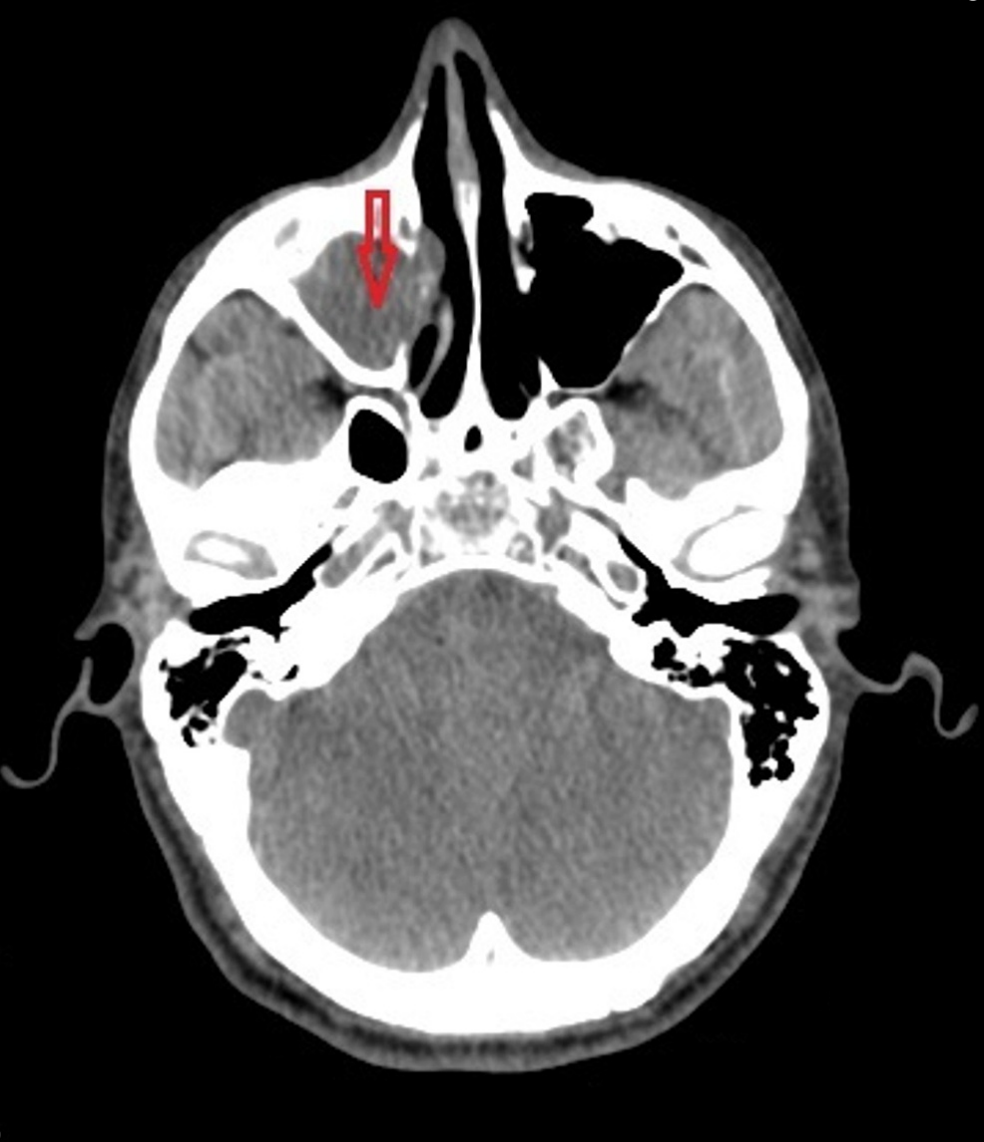
CT demonstrating opacified right maxillary sinus.

**Figure 2:**
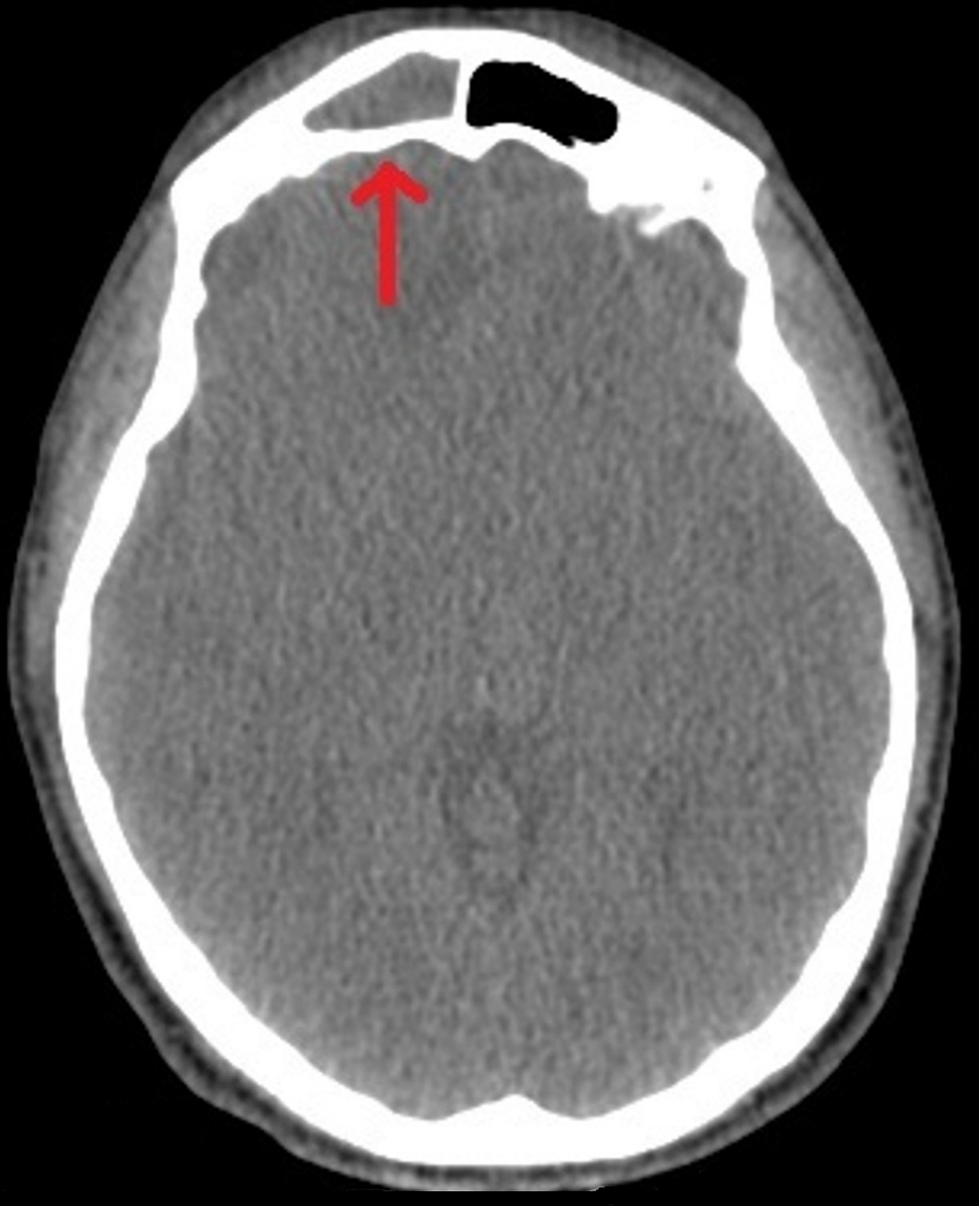
CT showing opacified right frontal sinus.

The CT scan that was performed revealed findings only in the right maxillary, ethmoid, and frontal sinuses but not in the parenchymal brain (Figures 1, 2). Concerning the laboratory results, it was found a leukocytosis of over 20,000 per cubic millimeter of blood, elevated levels of C-reactive protein (CRP) and erythrocyte sedimentation rate (ESR), and mild electrolyte disturbances. Because of high suspicion for meningitis or encephalitis, the patient was transported by ambulance to our university hospital for further investigation and treatment.

 

Firstly in the neurology department, an intravenous combination of ceftriaxone, clindamycin, and acyclovir was given along with antiepileptics. However, for the following three days, there was no response to the antibiotics. On the contrary, the patient displayed dyspnoea and bradycardia but echocardiography did not demonstrate any pathology. For the dyspnoea, a CT scan of the chest was executed, which revealed pleural effusion and therefore it was decided to modify the antibiotic medication with vancomycin and meropenem.

**Figure 3:**
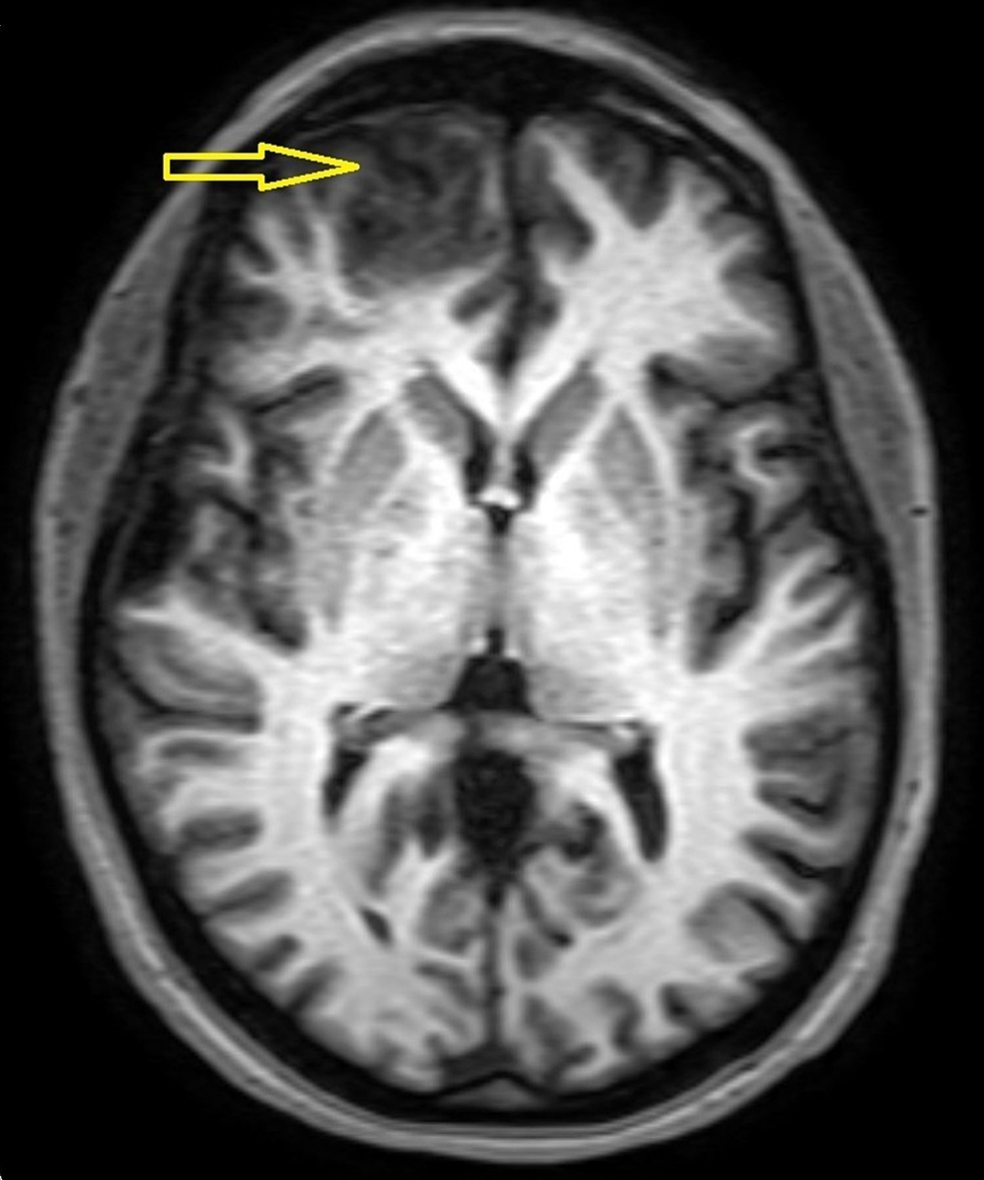
MRI brain indicating the presence of a parenchymal abscess in the right frontal lobe.

After 48 hours of treatment, the patient presented disorientation and abnormal behavior. The MRI of the brain that was performed, indicated the presence of a cerebral abscess in the right frontal lobe surrounded by oedema, which caused a slight displacement of the midline (Figure 3). In a new CT scan, shadows in the right frontal sinus accompanied by frontal bone thinning in the lower-posterior wall were observed.

A new evaluation was made by the otorhinolaryngologists who decided to proceed operatively. Endoscopically and under general anaesthesia, nasal polyps were detected in the nasal cavity, which were removed with the use of a flexible rhino endoscope. Then anterior ethmoidectomy and middle maxillary antrostomy were done along with drainage of pus from right sinuses. Ten days later, the fever was lower but still high and the leukocytosis was remaining. The patient started having disorientation while his headache was getting worse over days.

**Figure 4:**
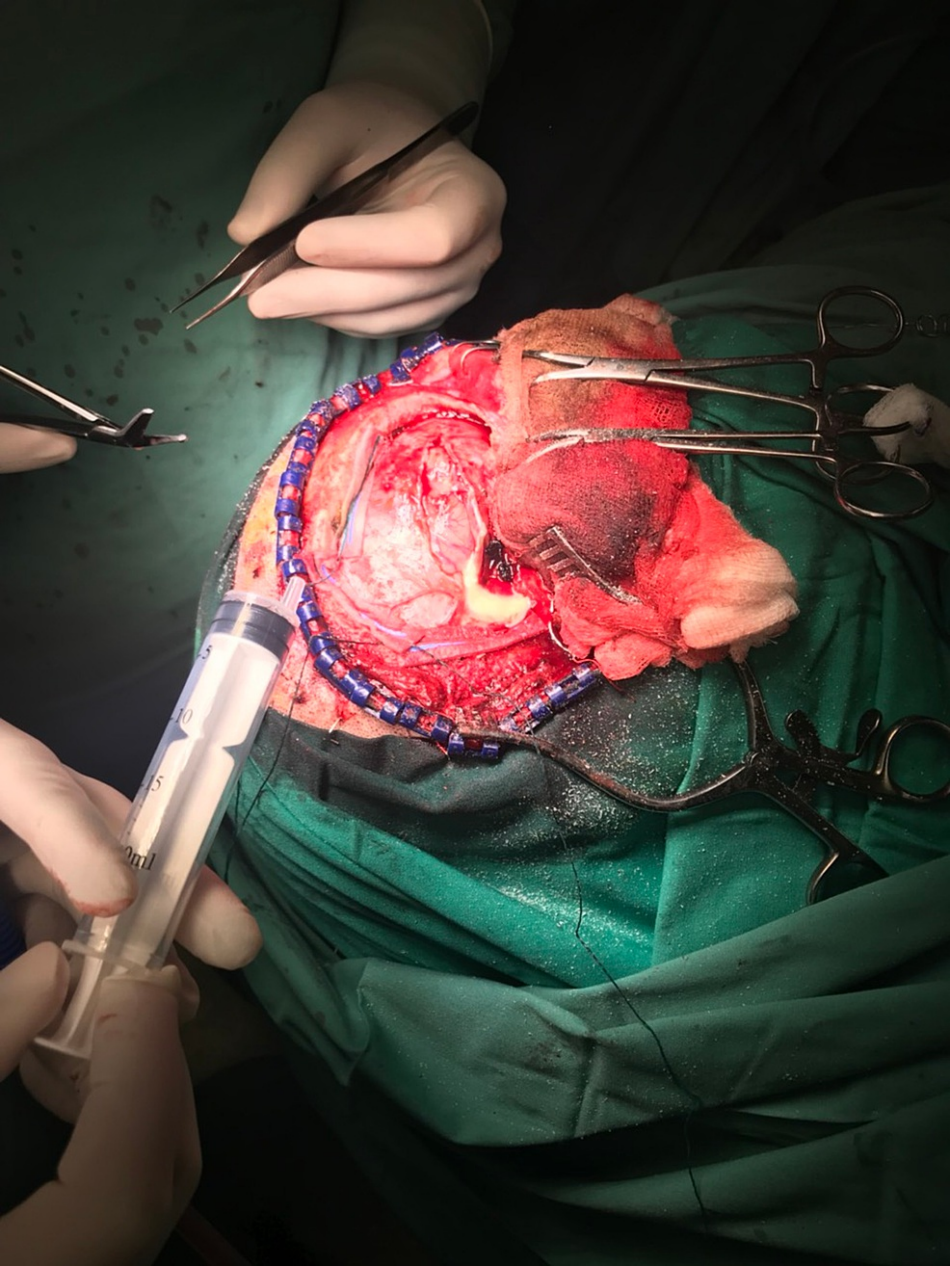
Pterional craniotomy for resection of the brain abscess.

Due to the unimproved clinical condition, neurosurgeons considered to proceed more drastically. Specifically, under general anaesthesia, a pterional craniotomy was performed and an open approach of the frontal and right temporal lobe was achieved (Figure 4). Thereafter, the brain abscess was found in the right frontal lobe and anterior part of the right temporal lobe. The purulent material and the surrounding abscess capsule were completely removed, with intend to eradicate the infectious sources and to decompress the brain parenchyma by lessening intracranial pressure. In addition, samples were taken from the abscess and sent for microbiological study for antibiotic sensitivity. Pus culture was consistent with alpha-hemolytic streptococcus that was sensitive to the antibiotics.

**Figure 5:**
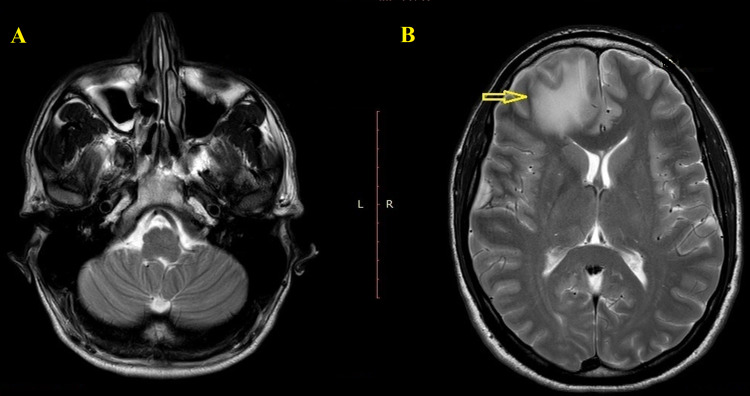
Six months postoperative MRI.

Subsequently, the patient was referred to the intensive care unit for constant care and close supervision for five days continuing intravenous antibiotics. The fever subsided within 72 hours. Neurological symptoms started resolving after 15 days and the patient was discharged nearly one month after the last operation presenting mild irritability, which was decreased gradually over the next two months. Six months later, an MRI was implemented where no signs of brain abscess were observed (Figure 5). At the time of the last follow-up, one year postoperatively, the patient was found to be totally asymptomatic.

## Discussion

Intraparenchymal brain abscesses are uncommon suppurative conditions that concern the central nervous system (CNS). In the majority of cases, they originate from contiguous structures with direct spread [1,6]. In our case, we report a brain abscess mainly in the frontal lobe which was the result of complicated sinusitis due to late diagnosis.

In general, orbital and dental infections or infections spreading from paranasal sinuses tend to affect mostly the frontal lobe whereas those from the middle ear are related mainly with abscesses in the temporal lobe [6,7]. Parenchymal abscesses from complicated sinusitis are frequently diagnosed at ages between 10 and 30 years old [6]. Their incidence in patients with acute or chronic sinusitis reaches almost 4% [8]. They usually develop through a pattern of early cerebritis progressing to encapsulation [9]. During this encapsulation phase, seizures may often occur [9] similar to our reported patient.

The widespread availability of CT has played an important role in the early diagnosis of brain abscesses contributing along with other factors, to the reduction of mortality from about 50% in the pre-CT period to less than 10% nowadays [10,11]. CT (with and without intravenous contrast enhancement) is considered the imaging method of choice not only for early diagnosis but also for surgical planning and accurate follow-up [11]. The abscess is usually depicted as a hypodense lesion with a complete rim of contrast enhancement surrounded by parenchymal oedema [12]. However, in our patient, the initial CT that was performed under intravenous contrast was not helpful for the diagnosis of the purulent parenchymal entity. Our suspicion was only confirmed by the MRI findings.

MRI is more sensitive in detecting early stages of abscess constitution or other alterations as compared to CT [12]. In cases of an incomplete capsule, CT hardly reveals the presence of capsulation, which can be observed in MRI. Moreover, MRI prevails in the exact localization and determination of special characteristics of such lesions. In particular, regarding the MRI, brain abscess is demonstrated like a lesion with a hypodense signal on T1-weighted and hyperdense signal on T2-weighted images, with a ring of enhancement surrounding the abscess and restricted diffusion on diffusion-weighted images [12-14].

It is indisputable that imaging modality plays a vital role as a diagnostic tool for patients with suspected complications. However, it should always be combined with relevant laboratory exams, patient’s medical history, and laryngological-neurological clinical evaluation in order to determine multiple-site involvement and guide the management of brain abscess resolution.

Depending on size, site, and numbers of suppurative intracranial space-occupying lesions, the patient may present symptoms that vary from headache and pyrexia to severe neurological dysfunction and epileptic seizures [15]. The commonest bacteria that have been isolated in brain abscesses caused by complicated otorhinolaringeal infections are anaerobic pathogens (Streptococci and Bacteroides spp amongst others) and aerobic gram-negative rods, such as *Morganella morganii* [16,17].

Concerning the medical management, apart from cases where conservative treatment could be sufficient, such as small lesions (<2.5-3 cm in diameter) with a known causative organism and no effect on neurological status, or unavoidable like in high-risk operative patients, in most cases, neurosurgical intervention is required [11-12]. From antibiotics, the empiric combination of vancomycin, metronidazole, and third-generation cephalosporin (ceftriaxone) covers a wide range of anaerobes and gram-negative aerobes. Even though the antibiotic treatment could be modified according to the isolated organisms from the microbial culture [11-12]. Commonly, young patients respond better to conservative treatment [15]. Interestingly, the role of steroids in brain abscesses is still controversial, as, on the one hand, they might be beneficial for patients with severe cerebral edema but on the other hand, they are considered as obstructing factors for penetration of antibiotics into the abscess area [12,15].

In deteriorating, life-threatening cases, which do not respond to conservative treatment, surgery is indicated. Surgical treatment includes aspiration which can be executed stereotactically or as an open procedure, and complete excision of the purulent contents and surrounding abscess capsule through open craniotomy [12,18-19]. A lower rate of recurrence is reported when extensive resections are performed [12,15]. Poor prognostic indicators which may increase the mortality rate include delayed diagnosis, severe focal neurological deficits, rapid progress of the disease, and intraventricular rupture [11,20]. In parallel, poorer outcome has been observed in infants and elderly persons [20].

Successful medical management requires prompt recognition, appropriate, broad-spectrum antibiotics, and surgery if needed. The final clinical outcome will be the result of the multifactorial approach of neurologists, clinical microbiologists, radiologists, otorhinolaryngologists, and neurosurgeons.

## Conclusions

Conclusively, even rare because of widespread use of antibiotics, intracranial complications of sinusitis may result from the indirect spread of frontal sinusitis. Early application of the appropriate imaging method and aggressive therapy to eliminate the infectious process including surgical intervention is essential to prevent potential long-term disabilities or even death.
